# Reinforcement learning optimisation for graded metamaterial design using a physical-based constraint on the state representation and action space

**DOI:** 10.1038/s41598-023-48927-3

**Published:** 2023-12-09

**Authors:** Luca Rosafalco, Jacopo Maria De Ponti, Luca Iorio, Richard V. Craster, Raffaele Ardito, Alberto Corigliano

**Affiliations:** 1 Department of Civil and Environmental Engineering, Politecnico di Milano, p.za L. da Vinci 32, 20133 Milano, Italy; 2 Department of Mathematics, Imperial College London, 180 Queen’s Gate, SW7 2AZ London, UK; 3 Department of Mechanical Engineering, Imperial College London, Exhibition Road, SW7 2AZ London, UK; 4 Umi 2004, Abraham de Moivre-CNRS, Imperial College London, SW7 2AZ London, UK

**Keywords:** Engineering, Mathematics and computing

## Abstract

The energy harvesting capability of a graded metamaterial is maximised via reinforcement learning (RL) under realistic excitations at the microscale. The metamaterial consists of a waveguide with a set of beam-like resonators of variable length, with piezoelectric patches, attached to it. The piezo-mechanical system is modelled through equivalent lumped parameters determined via a general impedance analysis. Realistic conditions are mimicked by considering either magnetic loading or random excitations, the latter scenario requiring the enhancement of the harvesting capability for a class of forcing terms with similar but different frequency content. The RL-based optimisation is empowered by using the physical understanding of wave propagation in a such local resonance system to constrain the state representation and the action space. The procedure outcomes are compared against grading rules optimised through genetic algorithms. While genetic algorithms are more effective in the deterministic setting featuring the application of magnetic loading, the proposed RL-based proves superior in the inherently stochastic setting of the random excitation scenario.

## Introduction

The reduced power requirements of recent small electronic components^1^ makes energy harvesting solutions a realistic alternative to conventional chemical batteries, allowing for clean and low-cost autonomous devices^2^. Several solutions have been developed for harvesting energy from elastic and acoustic waves, one of the most ubiquitous and accessible energy sources. Acoustic and elastic energy is transduced into electricity through electromagnetic, electrostatic and piezoelectric mechanisms^3^ or, alternatively, through magnetostriction and electroactive polymers^4,5^. Among these transduction mechanisms, piezoelectric materials are usually preferred thanks to their large power densities and ease of application^6^. The basic mechanism of piezoelectric energy harvesting is the direct piezoelectric effect converting the deformation of the host structure due to mechanical or acoustic vibrations to an electrical potential^7^.

**Figure 1:**
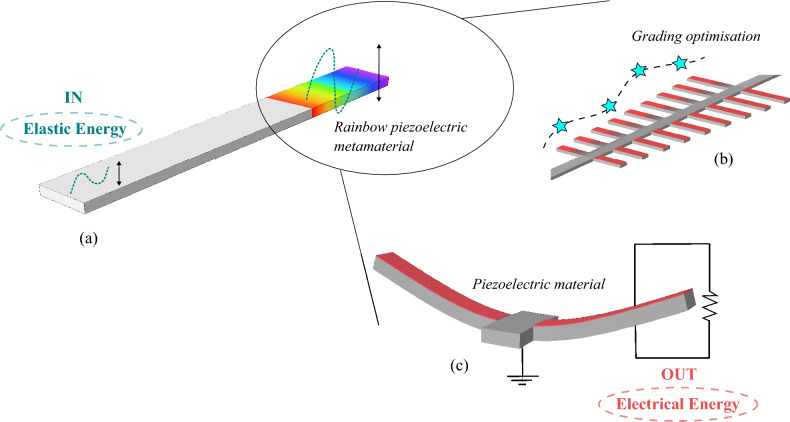
Schematic of the device used for energy harvesting and the optimisation procedure. (**a**) Elastic waves can be confined and amplified by leveraging graded metamaterials, i.e. by spatially varying the medium effective properties. Array guided waves slow down as they transverse the array with different frequency components localising at specific spatial positions, resulting in the so-called rainbow effect. This system, the rainbow metamaterial, can be physically obtained by attaching resonators to an elastic waveguide (**b**). To transduce elastic energy into electrical energy, we complement each resonator with piezoelectric materials, that are then connected to a resistor for collecting energy (**c**). As the system is based on the rainbow effect and enriched with piezoelectric insertions, we denote it as a *rainbow piezoelectric metamaterial*. The RL optimisation procedure changes the grading profile, here represented through a reduced set of variables (cyan stars), in order to optimise the total transduced energy of the array.

In an attempt to enhance energy harvesting capabilities, metamaterial based structures are increasing in popularity, thanks to their advanced performance in wave control and manipulation^8,9^. They often rely on local resonance for sub-wavelength control of waves, enabling the design of compact devices^10^. Since such systems already contain a collection of resonators, the inclusion of vibrational energy harvesters is straightforward leading to truly multifunctional meta-structures combining vibration insulation with harvesting^11–13^. To reduce wave scattering induced by impedance mismatch and simultaneously increase the broadband capabilities, graded designs have been proposed. The term *graded* is adopted to denote a smooth variation of a particular parameter of the local resonators along space (conventionally the resonant frequency), creating local band-gaps to control wave propagation. Graded metamaterials are thus able to manipulate waves by confinement over some spatial region along the structure, enabling wideband vibration attenuation and simultaneous energy harvesting^14–17^. In Fig. 1, we exemplify the idea of combining the use of the piezoelectric effect with a metamaterial based structure employing a graded array of resonators.

Different grading profiles have been studied for energy harvesting^17,18^, spatially programmable trapping or mode conversion^19,20^. High frequency homogenisation was employed to set the gradient function in graded metamaterials^21^. A general procedure for grading optimization (both in terms of frequency and spacing) has also been proposed^22^. The procedure, based on reinforcement learning (RL), has been developed to maximise the mechanical energy confinement of a graded metamaterial under monochromatic input. Whilst the solution obtained provides relevant insights on the physics of graded metamaterials, the relation between grading and energy harvesting efficiency remains an unsolved problem. Moreover, most of the studies on metamaterials consider ideal input sources, neglecting the effect of realistic noisy signals. In this paper, we define a RL procedure for the optimisation of graded metamaterials for energy harvesting under general loading conditions, here exemplified for the common cases of magnetic loading or random vibrations. Other works treating design optimisation as a Markov decision process (MDP) solvable via RL were proposed for phononic crystals for matching^23,24^ or maximising band-gaps^25^, and for acoustic metamaterials to minimise wave scattering^26^. The combination of MDP and RL was also employed in structural optimisation^27,28^, material design^29,30^, optics^31^, and chemistry^32^. Attempts to improve the computational efficiency of RL-based design optimisation were done by exploiting transfer learning to accelerate the design synthesis starting from data regarding existing bio-structures^33^; and by using convolutional neural network to generate optimisation initiation points closer to the final outcome^26^.

A RL-based approach has been preferred to gradient-based optimisation because RL does not require continuity and low-modality throughout the design space^34^, and because RL does not require the functional relation between the design parameters and the objective function which is hard to obtain when there are discontinuities with respect to the design parameters. Alternative optimisation methods such as particle swarm usually suffer from the difficulty of imposing constrains on the design parameters^35^. To assess the performance of the proposed RL-based procedure, a comparison has been made with genetic algorithms (GA)^36^, a state of the art metaheuristic method for nonlinear optimisation. When the optimisation problem is defined in a deterministic setting, GA have proved to be more effective and less computational demanding than the RL. However, when a stochastic setting is considered, the proposed RL-based procedure has notably outperformed GA.

While physics-informed machine learning (ML) has emerged as an effective and elegant approach to solve regression problems^37^, the combination of physical knowledge and RL has only been explored recently. For example, physical considerations were exploited to provide meaningful and synthetic representations of the studied system^38^, and governing equations were used to inform policy search and model learning^39^. Here, the design space is constrained on the basis of the physical understanding of wave propagation in a locally resonant system. Specifically, the dimensionality of the design space is reduced to get configurations possibly leveraging the rainbow effect^14,15,40,41^, i.e. spatial signal separation depending on frequency.

The innovative aspects of this paper concern both the methodology and the application. On the methodological side, the knowledge of the physics of the problem has been exploited to constrain the state representation of the system and the action space. At variance with what proposed in^22^, we modified the interpolation rules, used to reduced the dimensionality of the state of the system, on the basis of considerations on the learning of the RL agent, in this way greatly benefiting its training. Such considerations are general and can be applied to other design optimisation problems. On the application side, the optimisation of graded metamaterials for energy harvesting under a realistic input has been considered for the first time. The design we propose is fully compatible with micro fabrication paving a path towards the development of ultra-wide band (UWB) micro-electro-mechanical systems (MEMS)^42^ for energy harvesting using metamaterials.

## Results

**Figure 2:**
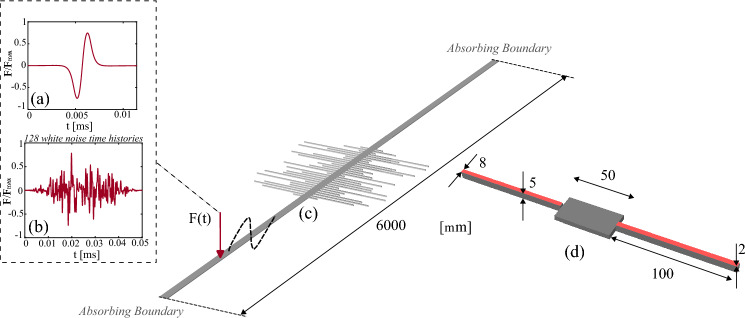
Geometry of the waveguide, and of the resonator array, for the reference random configuration. Distances are in micrometers. Each resonator is equipped with a piezoelectric patch. Two types of input are considered: (**a**) magnetic loading and (**b**) random excitation. Flexural waves, created by means of an out-of-plane force, propagate along the elastic waveguide and reach the array of resonators, while spurious reflections are avoided by imposing absorbing layers using increasing damping (ALID) (**c**). Detailed view of the unit cell, endowed with piezoelectric patches (**d**).

Dealing with energy harvesting at the microscale, one of the greatest limitations is the enormous mismatch between the frequencies at which the energy content of the environment is available and the MEMS natural frequency^43,44^. For this reason, frequency up-conversion (FuC) techniques have been developed to enable MEMS excitation at low frequencies^45^. Among different FuC techniques, a privileged position is occupied by the magnetic interaction via permanent magnets^46^, consisting of exploiting a magnetic field to generate an impulsive phenomenon on a piezoelectric transducer without any contact. An optimisation of the graded metamaterial for this kind of input is addressed here. As the theoretical evaluation of the magnetic force is a complex operation, out of the scope of the present paper, we use the simplified formulation developed by Akoun and Yonnet^47^, which demonstrated good experimental prediction for cubic magnets in Neodymium–Iron–Boron alloy^46^. In particular, the definition of the magnetic force is done by evaluating^47^: the magnetisation vectors; the magnetic permeability of vacuum; a few coefficients whose definition depends on the dimension of the two magnets and on the force direction. The magnetic loading is depicted in Fig. 2, alongside a second loading scenario that mimics the excitations to which the device is subjected under random vibrations. Specifically, 128 different time histories, each featuring a frequency spectrum within 0.1 and 2 MHz, are considered to account for the random nature of the vibrations. The use of many load histories avoids the trivial matching of single excitation spectrum peaks; from a numerical point of view, this random loading is generated through a white noise shaped by a Gaussian pulse.

In Fig. 2, we show the geometry of the metamaterial and the resonator configuration that is employed to assess the increase in energy harvesting possibly enabled by grading the resonators’ lengths. A number $\text {N}_{\text {r}}=30$ of resonators have been considered, symmetrically placed to minimise possible torsional motions^17^. Both the waveguide and the resonators are made from silicon with Young’s modulus $E_{\text {al}}=160$ GPa and density $\rho _{\text {al}}=2330$ kg/m$^3$. Each resonator is covered by a piezoelectric patch with density $\rho _{\text {piez}}=7800$ kg/m$^3$, Young’s modulus $E_{\text {piez}}=59$ GPa, piezoelectric constant $d_{31}= -171 \times 10^{-12}$ C/N, and permittivity at constant stress ${\bar{\epsilon }}^S_{33}= 13.3 \times 10^{-12}$ C/(Vm). The cross sections of the aluminium and piezoelectric parts are $8{\times }5 \mu$m and $8{\times }2 \,\upmu$m, respectively. The waveguide is $L_{w}=6$ mm long, and it has cross section $50{\times }5 \,\upmu$m. Therefore, the cross sectional area and the moment of inertia relevant for the dispersion relation of a propagating flexural wave are $B_{\text {w}}=250.0 \,\upmu$m$^2$ and $I_{\text {w}}=520.8 \mu$m$^4$, respectively. The resonator spacing is set to $5.5\, \upmu$m to enable resonator interactions at the sub-wavelength scale^48^. Alternative choices are not investigated due to the reduced impact of spacing on the dispersive properties of rainbow-based metamaterials^22^.

The mechanical response of the system is simulated through the finite element (FE) method by employing 304 Euler–Bernoulli beam elements to discretise the waveguide, and by associating a single degree of freedom (dof) to each resonator pair. Specifically, an equivalent lumped mechanical parameter system is employed to simultaneously model the mechanical response of the beam-like resonators and the effect of the attached circuit^17^. From the mechanical side, a certain mass $M_{\text {m}}$, damping $D_{\text {m}}$ and stiffness $K_{\text {m}}$ are associated to each resonator according to its first bending mode. An equivalent damping $D_{\text {e}}$ and stiffness $K_{\text {e}}$ is also introduced to account for the electrical side. Such quantities are determined through a general impedance analysis method^49^. The steps of the method are briefly described here; additional details can be found in^17,50^. First, the piezoelectric coupling term $\vartheta$ is computed on the basis of $d_{31}$ and of the distance between the centre of the piezoelectric layer and the resonator neutral axis. Second, the electro-mechanical coupling coefficient $\alpha$ and the piezoelectric capacitance coefficient $C_p$ are worked out from $\vartheta$, ${\bar{\epsilon }}^S_{33}$, and the geometric properties of the resonators. Last, the electrically induced damping $D_{\text {e}}$ and stiffness $K_{\text {e}}$ of the circuit are determined through the following relations:

1 $$\begin{aligned} D_{\text {e}}=\frac{\alpha ^2}{2}\text {Re}\left( Z_{\text {e}}\right) , \quad K_{\text {e}}=-\frac{\omega \alpha ^2}{2}\text {Im}\left( Z_{\text {e}}\right) , \end{aligned}$$

where $Z_{\text {e}}=\frac{1}{\omega C_p}$ is the equivalent impedance of the piezo-mechanical system; $\text {Re}\left( Z_{\text {e}}\right)$ and $\text {Im}\left( Z_{\text {e}}\right)$ accounts for the real and imaginary part of $Z_{\text {e}}$; $\omega$ is the resonance frequency of the considered resonator pair. The final damping and stiffness properties of each resonator pair are computed by summing mechanical and electrical counterparts, employing an iterative procedure to account for the consequent modification of $\omega$.

Two absorbing layers are used to avoid wave reflections^51^, thus minimising the operative conditions in which, typically, reflections at the waveguide extremities cannot be exploited due to the large substrate to which the device is attached. In the laboratory, such absorbing properties are achievable through acoustic black holes^15,52,53^. The length of the absorbing layers is set to 2.5 mm as it must be at least 4 or 5 times longer than the wavelength to be effective. Consequently, the exciting force, applied at $x=2.5$ mm, produces a left propagating wave immediately damped out by the absorbing conditions, and a right propagating wave interacting with the resonator array.

Wave propagation is performed in time with a time integration step set to $1.5 \times 10^{-8}$ s to correctly sample the vibration of each resonator pair and to match the Courant–Friedrichs–Lewy condition. The length of the analysis has not been fixed in advance considering that local interactions between resonators may require a very long time of observation^22^. Hence, the analysis is ended only if the harvested energy does not modify meaningfully for a sufficiently long time interval. The cumulative harvested energy is computed as in Eq. (2):

2 $$\begin{aligned} {\mathscr {E}}=\sum _{\text {n}_{\text {r}}=1}^{\text {N}_{\text {r}}}\int ^T_0\frac{1}{2} D^{\text {n}_{\text {r}}}_{\text {e}} \left( {\dot{u}}_{\text {n}_{\text {r}}}-{\dot{u}}^{\text {w}}_{\text {n}_{\text {r}}}\right) ^2(t) \text { d}t, \end{aligned}$$

where ${\dot{u}}_{\text {n}_{\text {r}}}$ is the velocity of the dof associated to $\text {n}_{\text {r}}$th resonator pair; ${\dot{u}}^{\text {w}}_{\text {n}_{\text {r}}}$ is the velocity of the dof of the waveguide to which the resonator pair is attached; *T* is the time length of the analysis.

The optimisation of the grading rule is carried out by a RL agent. The maximum length assignable to each resonator is roughly $300\, \upmu$m, the minimum is $10\, \upmu$m. The corresponding resonant frequencies are, respectively, 0.1 and 350 MHz. If one or more resonators are made shorter than $10\, \upmu$m, they are removed to avoid ill conditioning of the stiffness matrix.

**Figure 3:**
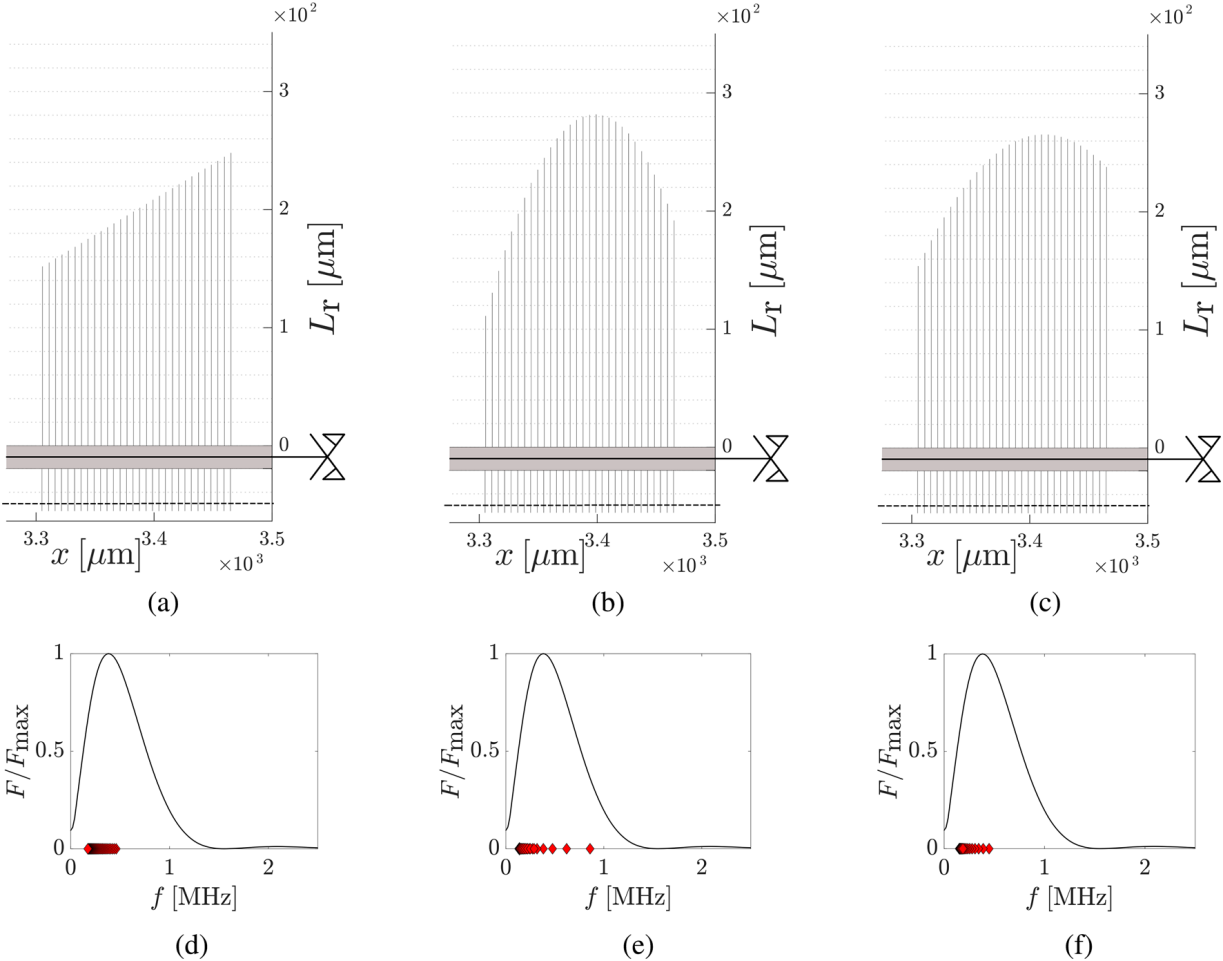
Magnetic loading: geometry and frequency characterisation of the resonators of the optimised configuration and of a linear grading amplifying the largest harmonic components of the loading. On top: geometry of (**a**) linear grading, (**b**) GA grading and (**c**) RL grading. On bottom: comparison between the first (equivalent) bending frequency of the resonator pairs, depicted by red diamonds, and the frequency spectrum of the magnetic loading for (**d**) linear grading, (**e**) GA grading and (f) RL grading.

For magnetic loading, the outcome of the RL-based procedure is reported in Fig. 3. The optimised RL configuration allows an increase in the harvested energy by 32.3% with respect to the random resonator arrangement depicted in Fig. 2 (that spans the same frequency range as the optimized array). In particular, the rainbow effect is enabled by the monotonic increase of the resonators’ lengths. Informing the RL agent of the physics of the problem facilitates this outcome, greatly reducing the variability of the optimisation process. Beyond the rainbow effect, resonators’ lengths are set to amplify the harmonic components of the loading that bear the highest amount of elastic energy. Figure 3 reports 3 different rainbow configurations: linear (Fig. 3a), optimized using GA (Fig. 3b), optimized using RL (Fig. 3c). The linear case is reported and compared to the other two to give a baseline over the effectiveness of a naively monotonic array. The GA solution is reported to show the result suggested by a different optimization algorithm. The RL solution finally is the one reached by a SAC policy. Both GA and RL converge to a solution where a fast initial change of resonators’ lengths is optimal for the combined purpose of slowing down the passing wave and absorbing it. More interestingly the function defined is not monotonic and it searches for a maximum resonator’s length in the second half of the array. Finally, in the last segment both optimizers choose to shorten again the resonators, confirming that this final length’s decrease is more effective or at least equally effective with respect to a simple length increase (commonly implemented in rainbow reflection systems). These last resonators have the role of reflecting back into the first part of the array the leftover energy components of the wave that manage to get thought the absorbing array. Generally higher resonators are implemented because they implicitly generate a local resonance band gap that stop and reflect the wave backward. In order to see whether this result of lower resonators has merit or it is an artifact of the constrain space, we performed new analyses in which the resonators after the highest one in the array are forced to increase rather then decrease. Result showed a similar efficiency (decrease of 0.4%) with respect to the original case. Looking at the linear grading and the one optimised through GA, they are respectively 1.9% and 38.4% more efficient with respect to the random case of Fig. 3. This implies that, for magnetic loading, GA find a better configuration when put up against RL. GA outperform RL also looking at the computing time. Specifically, GA complete the optimisation procedure in 1 h and 18 minutes by performing 50 generations, while RL takes 3 h and 47 minutes to run 5000 episodes. Analyses have been performed on a workstation equipped with Intel®Core$^{\text {TM}}$, i9 CPU @ 3.6 GHz and with 32 GB RAM.

For white noise loading, the performance of the RL based optimisation has been evaluated again by comparing it to a linear grading and a GA optimised grading. The white noise spectrum always spans the frequency range between 0.1 and 2 MHz but the energy carried by each frequency components is randomized for a total of 128 different loads. Moreover, given that the input to the numerical analyses is a force that follows the white noise function, the energy component of each frequency is not constant with frequency. The relation between the work inserted in the system by an applied harmonic force *F* and its frequency content (in steady state regime) is ${\mathscr {L}}(f) \div f^{-5/2}$. The proof is based on the Green’s function governing the response to an harmonic force of an infinite waveguide^54^ ruled by the Euler–Bernoulli beam theory. The extended proof and its numerical validation are reported in the supplementary material. This denotes an intrinsic energy decrease at higher frequencies for the same applied force. For the linear grading the upper limit of 2 MHz is exceeded by the resonance of the first three resonator pairs with which the propagating waves interact.

**Figure 4:**
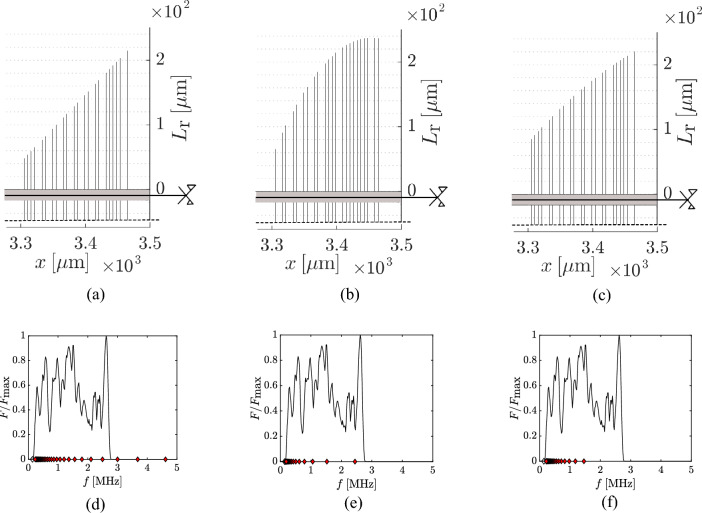
White noise loading: geometry and frequency characterisation of the resonators for the linear and optimised configurations. On top: geometry of (**a**) linear grading, (**b**) GA grading and (**c**) RL grading. On bottom: comparison between the first (equivalent) bending frequency of the resonator pairs, depicted by red diamonds, and the frequency spectrum of a possible random load for (**d**) linear grading, (**e**) GA grading and (f) RL grading.

**Figure 5:**
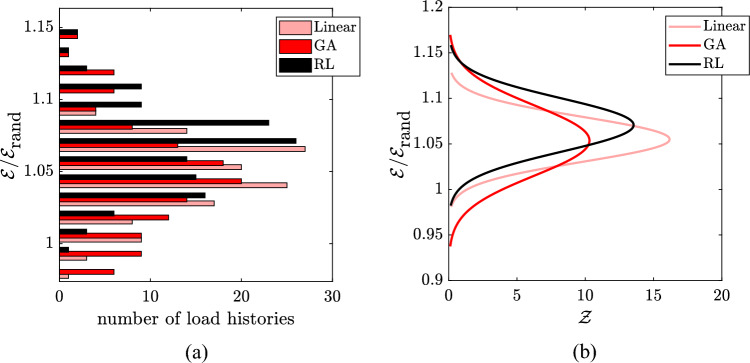
White noise loading: increase in the harvested energy for the linear configuration, and for the GA and RL optimised configurations with respect to the considered random resonator arrangement (rand). (**a**) Discrete distribution obtained for the considered random loads (128 load histories). (**b**) Gaussian approximation ${\mathscr {Z}}$ of the discrete distributions.

The outcome of the RL optimisation (depicted in Fig. 4c) for the white noise scenario is reported in Fig. 4 against the linear grading (Fig. 4a) and the GA optimised configuration (Fig. 4b). The RL optimised configuration outperforms both GA and the linear grading, as can be checked by looking at the harvested energy of each of the 128 loading scenarios reported in Fig. 5a. This result shows that it is beneficial to the graded array to have resonators’ heights that follow a monotonic increasing function and, more importantly, that are just concentrated on a smaller frequency range, where most of the energy of the input is concentrated. This means that the interval between 0.1 and 2 MHz is not fully covered. This can be in part attributed to the fact that the circuit damping broadens the bandwidth around the resonance, so a more distributed frequency range is actually absorbed. Looking at Fig. 5b, where ${\mathscr {E}}$ is the energy absorbed by all the resonators combined, a Gaussian distribution shows how much each configuration is able to generalise and be efficient for a wider range of loads. The comparison shows that the GA algorithm is actually more efficient at finding better performing configurations for a specific range of loads, while the RL optimiser finds a configuration that does not outperform the GA for most of the loads, but that can, in general, harvest more energy from all the possible load histories. Looking at the computing time, the duration of the optimisation processes run by the two algorithms is equal to the one detailed for the magnetic loading case, as the same number of generations (for GA) and of episodes (for RL) have been considered. It can be noted that constraining the design space has reduced the possible outcomes of the optimisation process, but it has not precluded obtaining unexpected configurations. Indeed, the finally obtained designs are not only of interest for fabrication, but also help in further understanding the physics of the problem.

## Discussion

We have shown the outcome of a RL-based optimisation procedure as applied to the design of a graded metamaterial under two realistic loading scenarios, the first considering magnetic loading as FuC mechanisms, the second mimicking the excitation due to a random source. The latter scenario has required us to evaluate the performance of the optimised design on a statistical basis by considering a set of excitation with different frequency content.

For magnetic loading, GA outperform the proposed RL-based approach, both looking at the performance of the system and at the computing time. On the contrary, for white noise loading, RL-based optimisation outperforms GA and the linear grading giving a baseline over the effectiveness of the optimisation algorithms. Such outcome is due to the stochastic optimisation setting featured by this latter load case.

The application of the RL-based optimisation procedure to the design optimisation of graded metamaterials for energy harvesting shows that: in one sense, physics can be effectively used to inform the optimisation procedure by constraining the design space; in the other sense, investigating the reason behind the decisions of the RL agent can improve the physical understanding of the problem highlighting working mechanisms different from the expected ones.

## Methods

**Figure 6:**
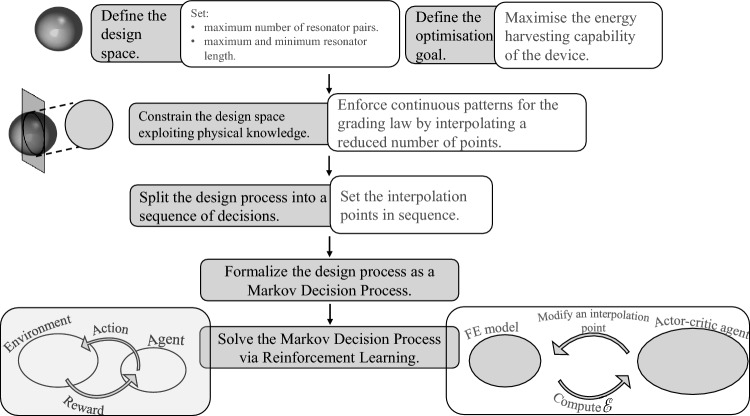
Methodology to design optimisation applied on graded metamaterials for energy harvesting. With gray background, the steps applied to formalise and solve a general design optimisation problem. With white background, the corresponding operations applied to the problem at hand.

The workflow of operations of the RL-based design optimisation is detailed in Fig. 6. A model-free actor-critic approach^55^, namely the soft actor critic (SAC) algorithm^56^, is used to maximise the optimisation target, here the amount of harvested energy. The RL agent takes actions according to a stochastic policy parametrised by a neural network (NN). The NN plays the role of a component of the agent termed actor. The policy is updated according to a second NN, modelling another component of the agent called critic. In turn, the critic is updated on the basis of the expected return computed in the environment, here the FE setting in which the response of the piezo-mechanical system is modelled. By assuming a finite number $\text {N}_{\text {t}}$ of states or, in other words, a finite number of decisions modifying the system configuration, the expected return $G_{\text {n}_{\text {t}}}$ at the current ${\text {n}}_{\text {t}}$th state is computed as:

3 $$\begin{aligned} G_{{\text {n}}_{\text {t}}} ={\mathscr {E}}_{{\text {n}}_{\text {t}}+1} +{\mathscr {E}}_{{\text {n}}_{\text {t}}+2} +\ldots +{\mathscr {E}}_{{\text {N}}_{\text {t}}}, \end{aligned}$$

where ${\mathscr {E}}_{{\text {n}}_{\text {t}}+1}$ is the harvested energy, see Eq. (2), for the $({\text {n}_{\text {t}}+1})$th state of the system. More details on the application of the SAC algorithm can be found in the literature^56^.

The use of SAC with respect to other RL algorithms is now motivated. Employing policy gradient methods is suggested to handle the stochastic optimisation setting required by the white noise load case. The actor-critic paradigm reduces the variance error in estimating the optimal policy^55^. Algorithms handling continuous action spaces have been considered. Looking for the best performance, we have tested the following algorithms on the white noise loading case: the deep deterministic policy gradient^57^ (DDPG), the trust region policy optimisation^58^ (TRPO), the proximal policy optimisation (PPO)^59^, and SAC. The latter has recorded the best performance. It is worth to mention that SAC enjoys the advantage of not requiring the tuning of the learning rate.

We have pointed out that the selected algorithm can handle continuous action space. In principle, a discrete action space could have been set by modifying the length of each resonator according to the tolerances of the manufacturing process. However, considering 30 resonators and a fabrication process tolerance of 0.15 $\upmu$m (consistent with the STMicroelectronics ThELMA®technology^42^) leads to an exploding number of possible designs. Consequently, a continuous action space and a continuous system state have been preferred, and the use of function approximators, namely NNs, is required instead of tabular approaches.

**Figure 7:**
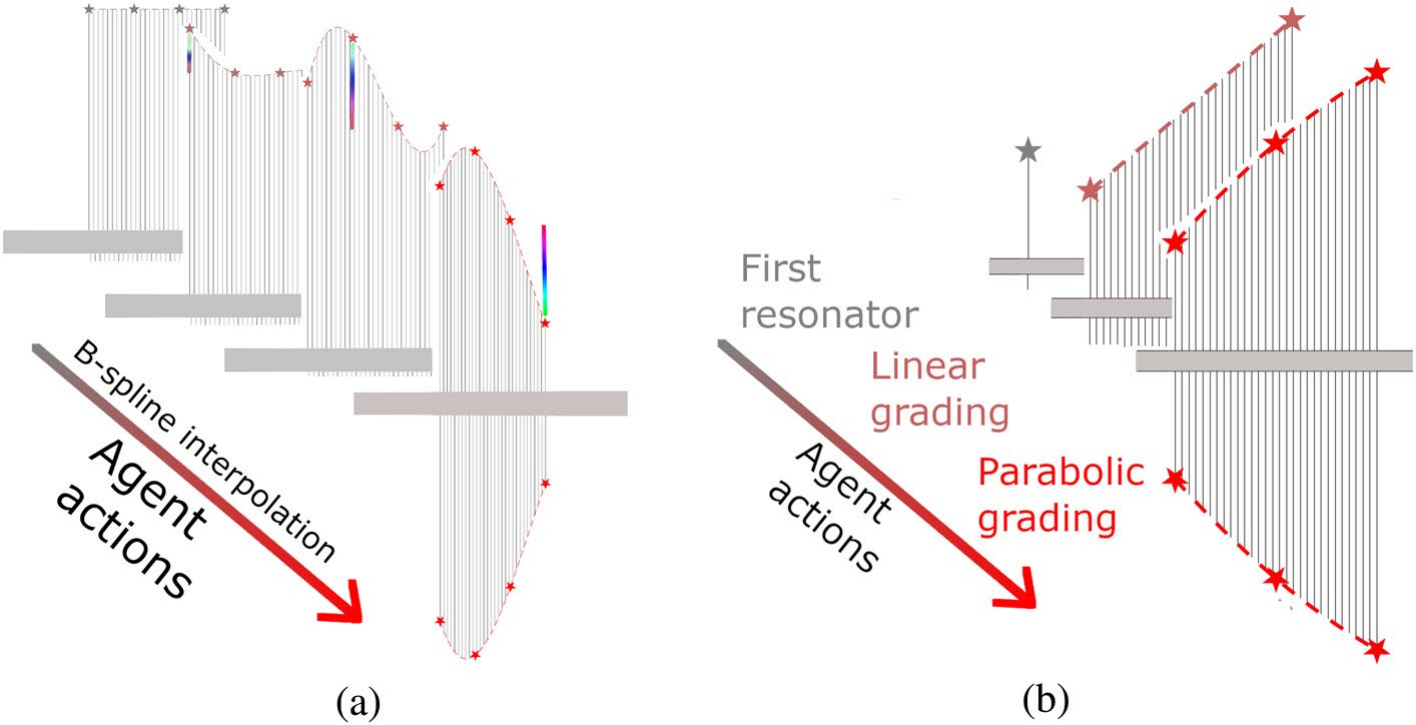
(**a**) Schematic representation of the decisions of the RL agent according to the previously proposed interpolation rule^22^ and (**b**) the current approach. The previous proposal made use of B-spline interpolation. In the current proposal, the interpolation points, depicted as stars, are placed at the start, at the end, at 1/2 of the resonator array. The first agent action places just the first resonator. The second action places all the resonators by setting the length of the last resonator and enforcing a linear grading rule. The third action sets the position of the middle interpolation point. Finally, the resonators’ lengths are set by the parabola passing through the three interpolation points.

As the automatic extraction through NNs of low-dimensional features informative of the system state is challenging in a RL setting, we have decided to provide an already dimensionality reduced vector as input in alternative of a vector detailing the lengths of each resonator. For this goal, we have introduced the interpolation rule reported in Fig. 7b as it limits the system space to just three dimensions. As a secondary outcome, we have reduced by 10 times (from 30 to 3) the number of states $\text {N}_{\text {t}}$ explored by the agent for each episode.

To reduce the dimensionality of the system state and of the action space, we have exploited the physical understanding of wave propagation in a local resonant system to exploit the rainbow effect. Specifically, resonators’ lengths are set according to an envelope defined by interpolating a reduced number of points, as done in our previous proposal^22^ in which B-spline interpolation was exploited. However, B-spline interpolation makes extremely challenging for the agent to foresee the effect of its decisions. As shown in Fig. 7a, every action of the agent, consisting in modifying one interpolation point, completely changes the lengths of each resonator, possibly altering the concavity and the ascending or descending behaviour of their envelope. The current proposal overcomes such difficulties. The first two actions, setting the lengths of the first and last resonator and a linear grading between them, enable to roughly define the operating frequency range of the resonators. The main effect of the third action is to control how fast the dispersion properties of the medium are modified allowing for a possibly parabolic final grading. The arrangement defined for one side of the device is then mirrored to get a symmetrical configuration.

In conclusion, we have demonstrated potential advantages of RL-based optimisation in designing graded arrays of resonators. Superior performances with respect to conventional optimisation procedures, e.g. genetic algorithms, have been demonstrated for inherently stochastic setting of random excitations. More in general, the greatest potential of the procedure is the ability to deal with stochastic optimisation. Possible limitations come from the large number of required model evaluations, implying a three times longer optimisation process with respect to genetic algorithms for the problem at hand. Such drawback becomes more severe by scaling the dimensionality of the problem. For this reason, a critical aspect of the method is represented by the definition of the physical-based constraint on the state representation and action space to avoid evaluating less promising designs, still allowing for unexpected outcomes as seen in the proposed applications.

Future work will act to refine the RL-based optimisation with additional interpolation points, as well as to include asymmetric configurations with further types of waves, e.g. longitudinal or torsional. Extensive experimental activity will be also carried out to validate the obtained numerical results. The proposed method could be applied to 2D or even 3D wave propagation problems for the optimisation of elastic lattices or frames. Finally, further extensions come from the ability to cope with not simultaneous, possibly competing, design goals thanks to the adopted MDP formalisation of the optimisation problem.

### Supplementary Information

Supplementary Information.

## Data Availability

The datasets analysed during the current study are available from the corresponding author on reasonable request.

## Code availability

Codes will be made available upon reasonable request.
