# Supplementary material for: Reinforcement learning optimisation for graded metamaterial design using a physical-based constraint on the state representation and action space
Source: Sci Rep. 2023 Dec 9;13:21836. doi: 10.1038/s41598-023-48927-3 (PMC10710405; doi:10.1038/s41598-023-48927-3)
Supplement: Supplementary file 1 — Supplementary Information. [file 41598_2023_48927_MOESM1_ESM.pdf]

This document forms the **supplementary material** to the manuscript
*"Reinforcement learning optimisation for graded metamaterial design using a physical–based constraint on the state representation and action space"*

# Proof of the relation between the energy inserted in a beam governed by the Euler–Bernoulli theory and the frequency content of the applied force

L. Rosafalco[1], J. M. De Ponti[1], L. Iorio[1], R. Ardito[1], R. V. Craster[2,3,4], and A. Corigliano[1,*]

[1]Dipartimento di Ingegneria Civile e Ambientale, Politecnico di Milano
[2]Department of Mathematics, Imperial College London
[3]Department of Mechanical Engineering, Imperial College London
[4]Umi 2004, Abraham de Moivre–CNRS, Imperial Collage London
[*]alberto.corigliano@polimi.it

Determining the relation between the work $\mathcal{L}$ done by a force $F$ applied to a beam and frequency content of $F$ allows to describe the amount of energy inserted into the system. The beam is governed by the Euler–Bernoulli theory. It is possible to demonstrate that such a relation is $\mathcal{L} \div f^{-\frac{5}{2}}$.

---

*Proof.*

Starting point of the proof is the governing equation of an infinite beam undergoing transverse motion and excited by a harmonic force $F(t) = \delta(x - \zeta)\mathrm{e}^{-ift}$ with frequency $f$ applied at the generic coordinate $x = \zeta$:

$$\frac{\partial^4 u}{\partial x^4} + \frac{EB_\mathrm{w}}{EJ_\mathrm{w}}\frac{\partial^2 u}{\partial t^2} = \frac{1}{EJ}\delta(x - \zeta)\mathrm{e}^{-ift}, \tag{1}$$

where: $\delta$ is the Dirac's delta; $J_\mathrm{w}$ is the moment of inertia of the waveguide; $B_\mathrm{w}$ is the cross sectional area of the waveguide; $E$ is the Young's modulus; $\rho$ is the material density.

The beam response in space and time is governed by the Green's function, see [**?**], here reported:

$$u(0,t) = \frac{1}{4\gamma^3 EJ_\mathrm{w}}\left(i\mathrm{e}^{i(\gamma(x-\zeta)-ft)} - \mathrm{e}^{\gamma(x-\zeta)-ift}\right), \tag{2}$$

with $a^2 = \frac{EJ_\mathrm{w}}{\rho B_\mathrm{w}}$, and $\gamma^2 = \frac{f}{a}$

At $x = \zeta$, the displacement $u(\zeta, t)$ reads:

$$u(\zeta,t) = \frac{(i-1)a^{\frac{3}{2}}\mathrm{e}^{-ift}}{4EJ_\mathrm{w} f^{\frac{3}{2}}}. \tag{3}$$

By applying the definition of work, the following relation is obtained:

$$\begin{aligned}\mathcal{L}(t) = F(t)\cdot u(\zeta,t) &= \frac{1}{EJ}\delta(x-\zeta)\mathrm{e}^{-ift}\frac{(i-1)a^{\frac{3}{2}}\mathrm{e}^{-ift}}{4EJ_{\mathrm{w}}f^{\frac{3}{2}}}\\ &= \delta(x-\zeta)\frac{(i-1)a^{\frac{3}{2}}\mathrm{e}^{-i2ft}}{4EJ_{\mathrm{w}}f^{\frac{3}{2}}}.\end{aligned} \tag{4}$$

As further step, we switch from the time to frequency domain by applying the Fourier transform, obtaining:

$$\begin{aligned}\mathcal{L}(f) &= \int_{-\infty}^{+\infty}\mathcal{L}(t)\mathrm{e}^{-ift}\mathrm{d}t\\ &= \delta(x-\zeta)\frac{(i-1)a^{\frac{3}{2}}}{4EJ_{\mathrm{w}}}\left(-\frac{1}{i3f}\right)[\mathrm{e}^{-i3ft}|_{-\infty}^{+\infty}.\end{aligned} \tag{5}$$

Looking at the function $\beta(f) = \mathrm{e}^{-i3ft}|_{-\infty}^{+\infty}$, it is possible to verify that $|\beta(f)| \leq 1$.

Finally, the expression of the work in the frequency domain is written as the product of a first term independent on $f$ (in parenthesis), $\beta(f)$ (bounded in value), and $f^{-\frac{5}{2}}$:

$$\mathcal{L}(f) = \left(\delta(x-\zeta)\frac{1-i}{i}\frac{a^{\frac{3}{2}}}{12EJ_{\mathrm{w}}}\right)\beta(f)f^{-\frac{5}{2}} \tag{6}$$

proofing the $\mathcal{L}(f) \div f^{-\frac{5}{2}}$ relation. *Quod erat demonstrandum.*

---

A comparison of this theoretical relation with numerical results obtained through the Finite Element Method (FEM) is reported in the following figure. A perfect superposition between the two curves is observed, confirming the validity of the $\mathcal{L}(f) \div f^{-\frac{5}{2}}$ relation.

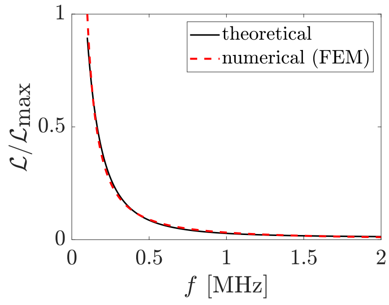


# References


[1] K. Graff, Wave Motion in Elastic Solids, Dover Books on Physics Series, Dover Publications, 1991.
